# Drug Development for Pulmonary Arterial Hypertension: Unleashing the Potential of Single‐Patient Studies Using Continuous Monitoring

**DOI:** 10.1002/pul2.70177

**Published:** 2025-10-15

**Authors:** Martin R. Wilkins, Sofia S. Villar, James Wason, Mark Toshner, Alexander M. K. Rothman

**Affiliations:** ^1^ National Heart and Lung Institute, Imperial College London London UK; ^2^ MRC Biostatistics Unit University of Cambridge Cambridge UK; ^3^ Papworth Trials Unit Collaboration Royal Papworth Hospital NHS Foundation Trust Cambridge UK; ^4^ Population Health Sciences Institute Newcastle University UK; ^5^ Department of Medicine University of Cambridge Cambridge UK; ^6^ Sheffield Pulmonary Vascular Disease Unit Royal Hallamshire Hospital, Sheffield Teaching Hospitals NHS Foundation Trust Sheffield UK; ^7^ Department of Clinical Medicine The University of Sheffield Sheffield UK

In the late 1940s, the US Air Force recorded an unacceptable number of crashes and emergency landings, prompting a formal enquiry. It was resolved that neither the engine nor the pilot was at fault. Attention turned instead to the design of the cockpit, which was based on the ‘average’ airman from 1926. Could it be that the body proportions of airmen 20 years on had changed? A subsequent survey of over 4000 airmen collected 132 detailed measurements on each pilot [1]. From this list, 10 were chosen that seemed most relevant to cockpit design and the number of airmen who fell into the middle 30% range for *all* 10 was calculated. The answer was 0. Even with three measures, under 3.5% of airmen were ‘average′. The survey report concluded that “The tendency to think in terms of the ‘average man’ is a pitfall… it is virtually impossible to find an ‘average man’ in the Air Force population” [2]. A contemporaneous competition to find ‘Norma′, the average American woman, based on average body proportions came to the same conclusion for women; the ‘average woman’ is rare [3].

These stories are well known [4]. Yet we continue to rely on average measurements in a variety of situations, including when designing and interpreting the results from clinical trials. In drug development, this may cause us to miss an efficacy signal that exists in a subset of subjects in a study that fails to meet a pre‐defined average endpoint: perhaps stopping a promising candidate drug from progressing further. Or it might lead to pursuing a higher dose than is necessary for ‘responders′, to achieve a pre‐defined averaged measurement for the whole study group. Moreover, translation of the mean result from a randomized clinical trial (RCT) to the clinic is frequently disappointing. Valid estimates are difficult to come by and numbers will depend upon the indication but less than a half and perhaps lower than a third of patients likely respond to the licensed dose [5, 6]. Relying solely on average patient data to inform a dose and dose regimen to treat an individual patient is a pitfall analogous to that of designing a cockpit for the average US Air Force pilot.

A common approach to addressing variation in drug response is to conduct a subgroup analysis of a RCT and identify ‘responders’ (i.e. a subgroup that appears to derive benefit from the drug) based on dichotomizing the outcome; using a threshold measurement that is viewed as clinically meaningful. But this is predicated on the assumption that the recorded response for each patient is reliable and reproducible; if the response cannot be replicated, then our identification of ‘responders’ is insecure [7].

N‐of‐1 studies are a subset of cross‐over trials designed to measure the variation in response of a patient to a certain treatment given more than once and on separate occasions. The patient is the unit of observation; they act as their own control and reproduction of the response on reintroduction of the study drug provides confidence that the patient is a responder to the drug. This type of study has a long history, but it is under‐employed in medical research [5, 8]. One reason is that certain criteria need to be satisfied to allow a N‐of‐1 study design. These include a stable background to provide a data set for meaningful comparison, easily measurable endpoints that can be objectively repeated, interventions with a relatively short half‐life to allow for washout and cross‐over of treatment and a protocol that provides careful management of the changeover treatment periods.

The opportunity to evaluate a new treatment in a small number of patients makes the N‐of‐1 study of particular interest to rare conditions, such as pulmonary arterial hypertension (PAH). PAH meets many of the criteria. It is a chronic condition where new treatments are introduced to affected patients who are stable on background licensed treatments. Established clinical measures of response can be repeated [9]. But there is an ethical concern around the safety of drug withdrawal in PAH. While desirable in drug assessment from a regulatory standpoint, as a return of clinical measures towards baseline provides added information with respect to drug effect, clinicians are sensitised to relatively rapid changes in cardiopulmonary haemodynamics that follow reduced or missed doses of some short‐acting vasodilators (e.g. prostacyclins) [10].

Here the introduction of wireless devices to medicine is a potential ‘game changer′. In addition to wearables, implanted devices, such as pulmonary artery pressure monitors and heart‐rate activity recorders, can be used to capture daily haemodynamic information during changes in therapy [11, 12]. Implanted devices, unlike wearables, cannot be forgotten or discarded, although patient adherence to data uploads is necessary. Regular remote data acquisition is not subject to terminal digit preference or seasonal variability [13, 14] and offers a dual benefit: it allows for the establishment of a stable baseline before a new drug is introduced, and it enables close, continuous monitoring of patients as their therapy changes. While the current number of PAH patients with implanted sensors is small compared to patients with left heart failure, patient acceptance has been high [15], there have been no safety concerns around the devices and the emerging results showcase their value [11, 12]. The attributes of digital technology permitting the collection of precise high‐frequency longitudinal data at the individual patient level augments what can be achieved with traditional clinical outcomes, where multiple repeated endpoints are expensive, time consuming and resource intensive, and allows the implementation of high‐quality single‐patient designs in PAH [16].

A recent study re‐evaluating imatinib as a treatment for PAH exemplifies the detail that can be obtained from the use of implanted devices in a single‐patient study setting [17]. This study substantiated an exposure‐dependent improvement in cardio‐pulmonary haemodynamics, namely total pulmonary resistance, over a tolerated dose range, based not on a single reading average, as is the case in RCTs, but the 3‐day rolling average. By elucidating the temporal relationship of response to exposure, the study suggested an earlier haemodynamic effect than commonly thought possible, an insight helpful in planning the timing of endpoints in future studies, and one that could enable the design of a shorter, more efficient future RCT. The study also provided evidence of a gradual return of haemodynamic measurements to baseline after drug withdrawal, an observation that assuages concerns about the impact of occasional missed doses and could inform dosing frequency in clinical practice.

The reproducibility of the hemodynamic response was shown by repeated exposure in the same patient (Figure 1). The optimum number of cycles of treatment required to reliably assess within patient variation in response to a treatment depends upon a number of factors, including the effect size, the precision of the measurement, and the stability of the disease. Another consideration is the length of time needed to produce an effect and the length of the washout period. This time commitment can place a burden on the patient as well as having cost implications for funders. Of course, the N‐of‐1 cross‐over design is only feasible if the treatment effect wears off within a reasonable timeframe after dosing is stopped; but that is itself instructive, as if no return to baseline is observed on drug withdrawal, this may go a long way towards answering questions around whether a drug is disease modifying. Where there is a reversal of response, a second challenge may be sufficient to provide confidence in a patient′s responder status (i.e. responder or nonresponder) in the context of daily recordings from implanted devices.

**Figure 1:**
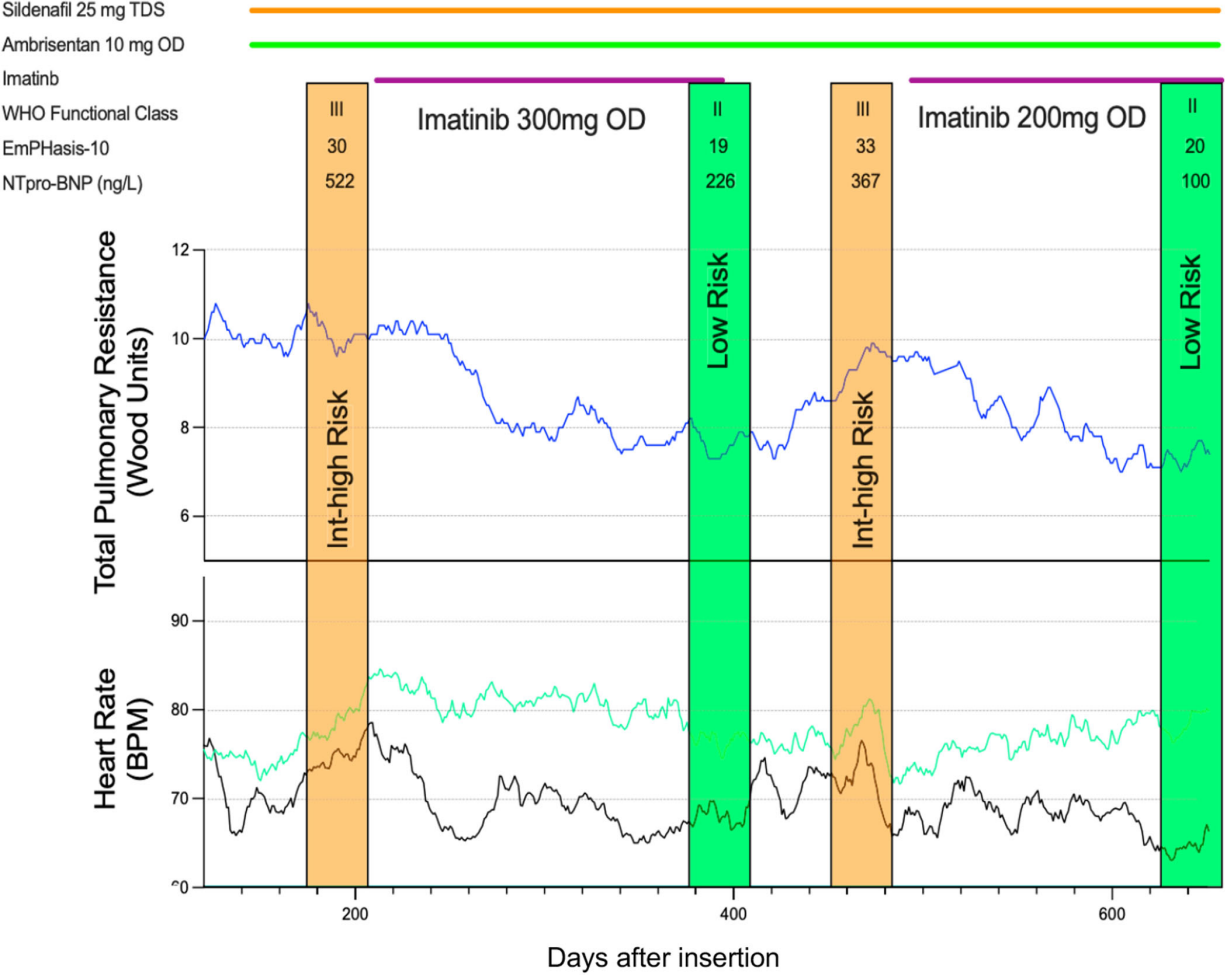
Rechallenging a patient with imatinib added to background therapy. Risk estimated according to ERS/ESC guidelines (modified from Ref [17]). Adapted with permission of the American Thoracic Society. Copyright © 2025 American Thoracic Society. All rights reserved.

Data from a series of N‐of‐1 studies of the same intervention can be combined to understand the potential average treatment difference. The primary methods for accomplishing this are a Meta‐Analysis of Individual Treatment Effects (where each N‐of‐1 trial provides an individual‐level treatment effect estimate to yield an average treatment effect for the studied population) and an Individual Participant Data (IPD) Meta‐Analysis using Hierarchical or Mixed Models (where hierarchical models are then used to simultaneously model within‐patient responses and between‐patient variability). In the presence of large heterogeneous treatment effects, a meta‐analysis of N‐of‐1 trials may offer efficiency gains over a traditional RCT approach to learn about effects in the population as opposed to the individual. By providing granular data and leveraging within‐patient comparisons to precisely quantify individual variability and overall effects, an N‐of‐1 series can identify and support clustering patients into distinct response subgroups.

But in considering N‐of‐1 studies with continuous monitoring for PAH, the idea is to complement rather than replace RCTs. Their optimal deployment is in early phase development, recognising that animal models are poor predictors of clinical efficacy and the best model organism is the human [18]. The N‐of‐1 study provides an early opportunity to conduct a proof‐of‐concept study in a small number of patients with a ‘fail early, fail fast’ strategy, the philosophy of recognising early when a drug is not effective or may even be harmful [19]; the drug can then be dropped before too many patients are exposed and resources can be re‐directed towards the next promising candidate, a priority for a rare condition. It can address efficiently the need to be inclusive of diverse populations; a positive response in just one patient from an underrepresented population is very powerful. As illustrated by the imatinib study, the N‐of‐1 design can accommodate novel trial designs [20] as well as instruct key design elements for larger trials, such as dose and dosing interval, truly meaningful effect sizes and optimal timeframes for treatment onset. This approach aligns with Project Optimus, a FDA initiative to reform and modernise dose selection and optimisation in oncology drug development by advocating for the identification of an optimal biologic dose [21]. The insights gained are invaluable for informing and refining the implementation of adaptive designs, allowing for more efficient mid‐trial adjustments and better resource allocation. Later on in the drug life cycle, N‐of‐1 studies have a place post‐marketing in that they provide the opportunity to monitor patients closely and evaluate different treatment combinations; rather than simply add new therapies to an ever‐increasing number of background drugs, once patients are stable, one or more drugs might be withdrawn and the impact carefully measured.

The answer to the ‘cockpit problem’ of the 1940s was an adjustable design that could be adapted to each individual airman. Ultimately, if wearables and/or implanted devices become more commonplace for patients with PAH, more patients may take part in their own N‐of‐1 study, allowing personalized treatment to target decisions [22]. This would align the management of PAH with the direction of travel for other chronic conditions, such as heart failure and diabetes. It parallels the broader necessity in modern drug development to employ experimental designs that can efficiently and robustly learn about population effects, while simultaneously facilitate the personalization of treatment.

## Author Contributions

The manuscript was drafted by Martin R. Wilkins and all authors contributed to the content and editing. It is not being considered elsewhere for publication.

## Ethics Statement

The authors have nothing to report.
